# Demographic and clinical characteristics of pulmonary arterial hypertension caused by schistosomiasis are indistinguishable from other etiologies

**DOI:** 10.1590/0037-8682-0418-2019

**Published:** 2020-02-07

**Authors:** Adriano Assis Mendes, Carlos Guilhermo Piscoya Roncal, Flávio Roberto Azevedo de Oliveira, Eugênio Soares de Albuquerque, Gustavo Henrique Belarmino Góes, Isabelle Cecília de Vasconcellos Piscoya, Dário Celestino Sobral

**Affiliations:** 1 Universidade de Pernambuco, Departamento de Hipertensão Pulmonar, Pronto-Socorro Cardiológico de Pernambuco, Recife, PE, Brasil.; 2 Universidade de Pernambuco, Departamento de Hemodinâmica, Recife, PE, Brasil.; 3 Universidade de Pernambuco, Departamento de Ecocardiografia, Recife, PE, Brasil.; 4 Universidade de Pernambuco, Faculdade de Ciências Médicas, Recife, PE, Brasil.; 5 Universidade de Pernambuco, Programa de Pós-Graduação em Ciências da Saúde, Recife, PE, Brasil

**Keywords:** Pulmonary hypertension, Schistosomiasis, Cardiopulmonary disease, Heart failure, Right ventricle

## Abstract

**INTRODUCTION:**

Pulmonary arterial hypertension (PAH) is a serious pulmonary circulation disease caused by several etiologies, including schistosomiasis. The present study retrospectively evaluated the clinical and hemodynamic characteristics of patients with schistosomal PAH (PAH-Sch) compared to those of non-Sch PAH patients (non-Sch PAH).

**METHODS:**

Patients treated at the Pronto-Socorro Cardiológico de Pernambuco and diagnosed by right cardiac catheterization were divided into PAH-Sch and non-Sch PAH groups. Their socio-demographic and clinical characteristics, N-terminal-pro B-type natriuretic peptide (NT-proBNP), and echocardiography and hemodynamic parameters were retrospectively reviewed.

**RESULTS:**

Among the included 98 patients (mean age, 45 ± 14 years; 68 women [69.4%]), we found 56 PAH-Sch and 42 non-Sch PAH. The age distribution was heterogeneous in the PAH-Sch group, with patients predominantly ranging from 50-59 (p <0.004). Dyspnea was the most common symptom, reported by 92 patients (93.8%), and commonly present for over two years prior to diagnosis. Clinical symptoms were similar in both groups, with no differences in functional class, pulmonary artery systolic pressure (p = 0.102), 6-minute walk test score (p = 0.234), NT-proBNP serum levels (p = 0.081), or hemodynamic parameters.

**CONCLUSIONS:**

Patients with PAH-Sch present clinical, laboratory, and hemodynamic profiles similar to those with PAH resulting from other etiologies of poor prognosis. PAH is an important manifestation of schistosomiasis in endemic regions that is often diagnosed late.

## INTRODUCTION

Pulmonary arterial hypertension (PAH) is a serious disease of the pulmonary circulation characterized by changes in the vascular wall, remodeling, vasoconstriction, and in situ thrombosis, with lesions primarily located in the pre-capillary segments of the pulmonary vasculature^1^ that can lead to a progressive increase in pulmonary vascular resistance (PVR), right ventricular failure, and early death. Hemodynamically, this syndrome is defined by a mean pulmonary artery pressure (MPAP) ≥25 mmHg and a PVR >3 Wood units with pulmonary capillary pressure ≤15 mmHg^2^.

Infection caused by *Schistosoma mansoni* currently affects approximately 207 million people in 75 tropical and subtropical countries, severely in 20 million, and approximately 779 million people are at risk of contracting this disease^3^. The prevalence of PAH associated with schistosomiasis (PAH-Sch) in patients with hepatosplenic schistosomiasis with hemodynamic confirmation ranges from 7.7-10.7%^4^ ^,^ ^5^. According to current PAH classification^6^, the schistosomiasis etiology is considered a part of Group 1.

The diagnosis of PAH has changed dramatically in recent years, as has its treatment, but early clinical recognition remains a challenge. The symptoms are often non-specific (i.e., cough, dyspnea, syncope, fatigue, and precordial pain) and are associated with significant morbidity and mortality, especially when identified in more advanced disease stages^7^ ^,^ ^8^.

**FIGURE 1:**
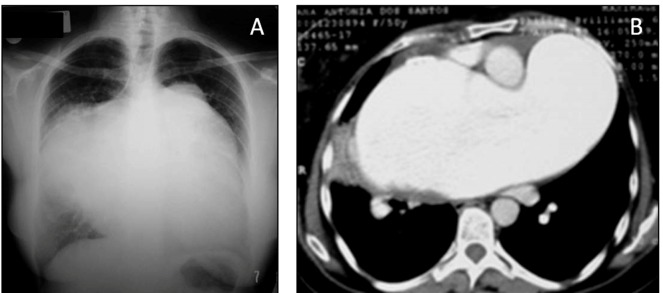
Chest X-ray (**A**) and computed tomography scan (**B**) of a patient with schistosomal pulmonary arterial hypertension and pulmonary artery aneurysm (“pulmonary artery ventricularization”).

Patients with PAH form a heterogeneous group, and it is necessary to distinguish the characteristics of PAH caused by schistosomiasis from other etiologies (Figure 1). Although there are some data regarding PAH-Sch, they primarily concern populations outside the known endemic zone. It remains unclear whether such findings can be extrapolated to populations living in endemic areas and manifesting the disease. Recent studies have indicated a possible improvement in survival using current therapies, alone or in combination, even in severe cases of PAH-Sch observed in endemic and non-endemic regions^9^ ^,^ ^10^. In the present study, we retrospectively assessed the clinical and hemodynamic characteristics of PAH-Sch carriers compared to the non-schistosomal PAH group (non-Sch PAH), considering the time period between symptoms and diagnosis in an endemic region of schistosomiasis in Northeastern Brazil.

## METHODS

Patients enrolled in the Pulmonary Hypertension outpatient of the Pronto-Socorro Cardiológico de Pernambuco (PROCAPE/University of Pernambuco) from January 2001 to August 2009 were selected for the current study. We retrospectively reviewed patient charts and extracted the demographic, clinical, laboratory, echocardiographic, 6-minute walk test (6MWT), and hemodynamic variables. The diagnosis of PAH was established when the MPAP was >25 mmHg, pulmonary capillary pressure <15 mmHg, and PVR >3U Wood, as assessed by right cardiac catheterization^2^ with a nitric oxide vasoreactivity test. All included catheterizations were from the moment of diagnosis. Patients were classified according to guidelines proposed at the World Symposium on Pulmonary Hypertension held in Nice in 2013. The diagnosis of schistosomiasis was based on abdominal ultrasonography with the presence of periportal fibrosis associated with the origin (area endemic for schistosomiasis), which may or may not be associated with previous treatment or positive screening for *Schistosoma* eggs by routine methods. Patients with alcohol-related hepatic parenchymal disease, metabolic diseases, hepatitis C or B, as well as those with chronic pulmonary diseases, chronic pulmonary thromboembolism, and patients with evidence of pulmonary venous capillary hypertension (capillary pressure >15 mmHg) at the time of diagnosis, were excluded. The 6MWT was performed in all patients according to the American Thoracic Society protocol^11^. Pulmonary arterial hypertension severity was assessed using the World Health Organization functional class (FC) adapted from the New York Heart Association^12^ . Laboratory tests were performed according to routine PAH protocols, as was the serum N-terminal-pro B-type natriuretic peptide (NT-proBNP) determination. We use transthoracic echocardiogram to estimate the pulmonary artery systolic pressure (PASP) by calculating the pressure gradient between the right ventricle and the atrium and applying the modified Bernoulli equation. A concomitant assessment of left ventricular function was performed to exclude the diagnosis of left ventricular failure^13^. This study was approved by the Human Ethics and Research Committee of the University of Pernambuco (CAAE 0116.0.106.000-09).

### Statistical analysis

Collected data were stored in an Excel database. Data were then exported to SPSS software version 19.0. To compare schistosomiasis and other etiologies (idiopathic, collagen, congenital, and human immunodeficiency virus [HIV]), the general characteristics, clinical complaints, physical characteristics, and hemodynamic variables were compared using the chi-square test or Fisher’s exact test (when necessary), in the case of categorical variables, and are presented as absolute (N) and relative (%) frequencies. For continuous variables, to determine whether the groups studied were homogeneous, the Student’s t-test was applied, and these variables are shown as absolute frequency (N) as well as mean ± standard deviation. A significance level of 5% was considered for all analyses.

## RESULTS

The study included 98 patients (mean age 45 ± 14 years) registered at the PROCAPE/UPE reference center with a diagnosis of PAH between 2001 and 2009 (Table 1). The majority were women (69.4%; n = 68), and the age variation was not statistically significant. The age distribution analyses indicated that the PAH-Sch group predominantly included patients in the fifth decade of life (Table 1). 

The most prevalent etiology was schistosomiasis in 56 patients (57.1%), followed by idiopathic in 19 (19.4%), collagen in seven (7.1%), congenital in ten (10.2%), and HIV in six (6.1%). Dyspnea was the most common symptom, reported by 93.8% (n = 92) of the study patients, and had been present for over two years in 43.3% of the study group. Table 2 presents descriptive statistics of the patient symptoms and the comparison between the patients with or without schistosomiasis. From physical examination, the most frequent finding was the presence of loud P2 (92.4%). Four patients (4.1%) were in FC I, 25 (25.5%) in FC II, 32 (32.7%) in FC III, and 37 (37.8%) in FC IV. There were no statistically significant differences between the PAH-Sch and non-Sch PAH groups (Table 1).

**TABLE 1:** Baseline demographic and hemodynamic clinical characteristics of the study groups.

General features	PAH-Sch	non-Sch PAH	Total	p* value
	N^†^	N^†^	N	
Age (years)*	56 (46 ± 13)	42 (42 ± 15)	98 (45 ± 14)	0.172
**Gender** ^†^				
Male	20 (35.7%)	10 (23.8%)	30 (30.6%)	
Female	36 (64.3%)	32 (76.2%)	68 (69.4%)	0.206
**Functional class** ^†^				
I	03 (5.4%)	01 (2.4%)	04 (4.1%)	
II	17 (30.4%)	8 (19.0%)	25 (25.5%)	
III	16 (28.6%)	16 (38.1%)	32 (32.7%)	
IV	20 (35.7%)	17 (40.5%)	37 (37.8%)	0.490
				
Echocardiogram (PASP, mmHg)*	56 (97.28 ± 27.23)	41 (88.76 ± 21.80)	97 (93.68 ± 25.31)	0.102
6MWT (m)*	56 (241.70 ± 126.70)	42 (211.10 ± 122.91)	98 (228.58 ± 125.38)	0.234
NT-ProBNP* (pg/mL)	43 (1444.23 ± 2202.45)	24 (2528.27 ± 2720.45)	67 (1832.54 ± 2437.25)	0.081
				
MPAP (mmHg)*	56 (60.80 ± 16.26)	42 (57.07 ± 19.58)	98 (59.20 ± 17.25)	0.291
MPRA (mmHg)*	56 (13.11 ± 5.98)	42 (13.57 ± 7.34)	98 (13.31 ±6.56)	0.731
PVR (U Wood)*	56 (48.31 ± 22.10)	42 (45.39 ± 29.70)	98 (2.58 ± 1.16)	0.578
CI (L/min/m²)*	56 (2.53 ± 1.15)	42 (2.65 ± 1.20)	98 (2.58 ± 1.16)	0.608
**S**_pa_ **O**_2_ *	56 (58.88 ± 13.67)	42 (63.14 ± 11.13)	98(60.70 ± 12.76)	0.102

**PAH-Sch:** schistosomal pulmonary arterial hypertension; **non-Sch PAH:** non-schistosomal pulmonary arterial hypertension; **PASP:** pulmonary artery systolic pressure; **6MWT:** 6-minute walk test; **NT-proBNP:** N-terminal-pro B-type natriuretic peptide; **MPAP:** mean pulmonary artery pressure; **MPRA:** mean pressure right atrium; **PVR:** pulmonary vascular resistance; **CI:** cardiac index; **S**_pa_ **O**_2_ **,:** oxygen saturation in the pulmonary artery. *N (mean ± SD); †N (%).

**TABLE 2:** Clinical complaints and physical examination of patients with pulmonary arterial hypertension.

Features	PAH-Sch		non-Sch PAH		Total		p* value
	N	(%)	N	(%)	N	(%)	
							
**Edema**	16	(41.0)	12	(42.9)	28	(41.8)	0.881
							
**Chest pain**	13	(34.2)	8	(28.6)	21	(31.8)	0.627
							
**Presyncope**	9	(23.7)	6	(21.4)	15	(22.7)	0.829
							
**Syncope**	10	(26.3)	7	(25.0)	17	(25.8)	0.904
							
**P2 hyperphonectically**	34	(89.5)	27	(96.4%)	61	(92.4%)	0.385
							
**Right ventricle B3**	6	(15.8)	4	(14.3)	10	(15.2)	1.000
							
**Jugular stasis**	8	(21.1)	10	(35.7)	18	(27.3)	0.186
							
**Hepatomegaly**	9	(23.7)	12	(42.9)	21	(31.8)	0.098
							
**Lower limb edema**	12	(31.6)	13	(46.4)	25	(37.9)	0.219
							
**Chance of pulmonary auscultation**	0	(0.0)	4	(14.3)	4	(6.1)	**0.028**
							
**Ascites**	0	(0.0)	4	(14.8)	4	(6.2)	**0.026**
							
**Cyanosis**	11	(28.9)	9	(32.1)	20	(30.3)	0.780
							

**PAH-Sch:** schistosomal pulmonary arterial hypertension; **non-Sch PAH**: non-schistosomal pulmonary arterial hypertension.

The average distance walked was 228.58 m (desvio padrão ± 125.38 m), and the median distance walked was 246.5 m. The analysis of variance test result indicated that that the mean 6MWT results varied according to the FC (p <0.001). At the time of diagnosis , the MPAP, measured by the hemodynamic study, and the PASP displayed a positive and significant correlation (r = 0.611, p <0.001).

The NT-proBNP results of FCs I and II compared to FCs III and IV in the general group presented a statistically significant difference (p = 0.001). These data indicated that natriuretic peptide values ​​tended to be lower in FC I or II than in FC III or IV. There was no significant difference in serum NT-proBNP levels between patients in the PAH-Sch and non-Sch PAH groups (p = 0.081). The right cardiac catheterization was performed at the time of diagnosis in all 98 patients, and there was no significant difference between the means of hemodynamic parameters in PAH-Sch and non-Sch PAH groups (Table 1). 

## DISCUSSION

This is the first study comparing the clinical and hemodynamic characteristics of patients with different forms of PAH in an area endemic for schistosomiasis. Overall, the characteristics of patients with PAH-Sch were similar to those of patients with PAH resulting from other etiologies. 

Among the 98 included patients with PAH, the prevalence of PAH-Sch was 57.1%, unlike other studies developed in a non-endemic region^14^ that showed a prevalence of 30%. This difference can be attributed to the fact that the referral center in the current study is located in a region where disease control remains unsatisfactory, with high morbidity/mortality rates, increasing social and economic costs to the region, and worsening patient quality of life^15^. In a United States registry of 578 patients, the prevalence of idiopathic PAH (IPAH) and connective tissue diseases (CTD) was 44% and 30%, respectively; in the French registry of 674 patients^17^, the prevalence of IPAH and CTD was 39% and 15%, respectively. However, in the present study, cases of IPAH and CTD comprised 19.4% and 10.2%, respectively, unlike other studies, which can be attributed to lack of recognition of the disease. In the French study^17^, the presence of PAH resulting from HIV was 6.2% versus 1.9% as shown by the REVEAL^18^ study. Six cases of HIV (6.1%) were identified in this study, although we believe that they are still underdiagnosed but similar to those previously reported in Brazil (4.5%)^19^. Mortality remains high even with specific therapies for PAH^20^ ^,^ ^21^. Further, women (69.4%) predominated, corroborating the higher incidence of PAH in the female sex, similar to the data from the French registry (65%) and the study by Rich et al. that reported a ratio of 1.7:1.0 between the sexes^17^ ^,^ ^22^. The mean age was 45 ± 14 years, while other studies indicated means of 48, 50, or 36 years^16^ ^,^ ^17^ ^,^ ^22^. Patients with PAH had a mean age of 56 years, suggesting late recognition of the disease in our region.

At the time of diagnosis, 70.5% of the patients were categorized as FC III or IV, similar to other studies that indicated rates of 80% and 75%, respectively^16^ ^,^ ^17^. Dyspnea was the most common symptom, affecting 93.8% of patients (n = 92). In 43.3% of cases, dyspnea was present for over two years prior to diagnosis, demonstrating a long symptomatologic period occurring in advanced stages of the disease. Rich et al.^22^ reported that dyspnea was present as an initial symptom in 60% of patients. The importance of dyspnea at the time of early diagnosis of PAH is that this is an important prognostic factor^7^, which makes it fundamental for identifying this pathology. In the REVEAL study^16^, the recognition of PAH at more than two years after symptom onset was 39.3%, which was very different from the registry in the current study, suggesting that our population presents additional confounding factors such as difficulty accessing public health services, lower purchasing power, presence of comorbidities, and even a lack of knowledge of the disease, among other factors that make an early diagnosis by a general practitioner difficult. The aim of identifying early-stage PAH is a possibility of initiating specific therapy to prevent the development of right heart failure^23^. Two symptoms to be highlighted in this study are pre-syncope and syncope, which occurred in 22.7% and 25.8% of patients, respectively. These symptoms occur in PAH in advanced stages of the disease, with signs of right heart failure and low output, demonstrating the severity of the disease in this group. In another study of 187 patients, syncope was present in 13% at the time of diagnosis^22^. 

Angina pectoris, another symptom of disease severity, was present in about 47% of patients with IPAH in a study of 187 patients; among these, etiology was not fully established in 22. Patients with PAH had few cardiovascular risk factors, since coronary atherosclerosis is unlikely as the cause of angina in these patients. Chest pain was reported by 31.8% of our patients. One of them presented spontaneous dissection of the marginal branch of the circumflex artery and was treated with a stent. Another with PAH-Sch presented with complete compression of the trunk of the left coronary artery, which was treated with a stent. The patient remained symptom free and showed good long-term outcomes, being the first case with this type of PAH-Sch treatment described in the literature^24^. Although chest pain is present in diverse clinical situations, it is always recommended that coronary angiography be performed in those with this symptom or who have significant dilatation in the large pulmonary branches^25^ ^-^ ^26^. The physical examination of patients with PAH is essential for the clinical diagnosis since 92.4% of the patients in our study presented loud P2 , while the right ventricular gallop was present in 15.2%, reflecting an increase in right atrial pressure similar to a study by Rich et al.^22^.

The 6MWT is an important tool for the prognostic evaluation of PAH. In the present study, we observed a mean distance of 228.58 m with no statistically significant intergroup difference. The disease severity in these patients is evident compared to reports from the French registry^17^ (329 m) and Miyamoto et al.^27^ stating lower survival in patients who traveled less than 332 m. Transthoracic echocardiography is undoubtedly the method of choice of screening for the diagnosis of PAH, since it is noninvasive, safe, and carries a lower financial cost. In the studied patients, PASP levels were quite high (mean 97.28 mmHg for PAH-Sch and 88.76 mmHg for non-Sch PAH). There was a positive and significant association between PASP and MPAP measured in a hemodynamic study (p <0.001) in our patients, similar to the results of previous studies^28^ ^-^ ^30^. Transthoracic echocardiography presents parameters of great importance for the evaluation of patients with PAH, including the severity of right ventricular dysfunction, right atrial enlargement, and pericardial effusion^31^ ^-^ ^32^, in addition to being useful to exclude other causes of PAH. Investigating the role of natriuretic peptides, Nagaya et al.^33^ evaluated a series of 60 patients with IPAH and demonstrated that BNP was correlated with HR, hemodynamic parameters of the right ventricle, and survival, functioning as an independent marker of mortality. Among the 43 patients with PAH-Sch in this study, the mean NT-proBNP was 1444.23 pg/mL, a value that was not significantly different from that of the non-Sch PAH group.

A study performed by Alonzo et al.^34^ reported that the independent hemodynamic variables mean right atrium pressure (mRAP), MPAP, and cardiac index were associated with a reserved prognosis. Oxygen saturation in the pulmonary artery is another relevant survival predictor^35^. In our study, patients with PAH-Sch and non-Sch PAH with reduced cardiac index, and elevated mRAP , MPAP, and PVR demonstrated the severity of this population at the time of diagnosis, as suggested by other authors^17^ ^,^ ^18^. Patients with non-Sch PAH had values ​​similar to those of the PAH-Sch group, but this difference was not statistically significant. Symptom severity, FC, 6MWT, NT-proBNP levels, and hemodynamic variables reflect the status of right ventricular failure, demonstrating the poor myocardial reserve of these patients and the disease severity. Thus, an early diagnosis is essential for the initiation of specific therapy and better patient prognosis^35^.

This study demonstrates the significant prevalence of PAH-Sch in our country, as well as its severity at the time of diagnosis, in patients of productive age and provides important implications for the region’s economy. However, many questions remain open, such as the actual number of asymptomatic PAH patients or the best epidemiological, clinical, or laboratory methods for early disease detection. The authors’ experience with 98 patients with PAH in this registry allowed for the correlation of several clinical and hemodynamic variables for a better understanding of this pathology. As there is no curative therapy, perhaps a high index of suspicion and knowledge of the clinical and hemodynamic findings described in this article may contribute to an increased number of diagnoses in less advanced stages.

The present study has some limitations. First, it is retrospective and observational. There is also the possibility of selection bias since it uses data from a single center. In contrast, all clinical, diagnostic (echocardiographic, hemodynamic, and computed tomography), and laboratory investigations were always performed by the same group of investigators from the beginning, thus reducing possible bias. Pulmonary arterial hypertension is an important manifestation of schistosomiasis in endemic regions. The diagnosis is usually made late but should be considered in patients with chronic dyspnea, as the clinical, hemodynamic, and laboratory presentations are similar to those of PAH resulting from other etiologies.
